# Changes in sexually transmitted infections-related sexual risk-taking among young Croatian adults: a 2005-2021 three-wave population-based study

**DOI:** 10.3325/cmj.2023.64.186

**Published:** 2023-06

**Authors:** Ivan Landripet, Ivana Božičević, Valerio Baćak, Aleksandar Štulhofer

**Affiliations:** 1 Department of Sociology, Faculty of Humanities and Social Sciences, University of Zagreb, Zagreb, Croatia; 2 School of Medicine, University of Zagreb, Zagreb, Croatia; 3 School of Criminal Justice, Rutgers University, Newark, New Jersey, USA

## Abstract

**Aim:**

To assess the prevalence and dynamics of risky sexual behaviors among Croatian emerging adults in the 2005-2021 period.

**Methods:**

Three surveys were conducted on large-scale national samples of young adults aged 18-24 in 2005 (N = 1092) and 18-25 in 2010 and 2021 (N = 1005 and N = 1210, respectively). The 2005 and 2010 studies were conducted with face-to-face interviews on stratified probabilistic samples. The 2021 study was conducted by computer-assisted web-interviewing on a quota-based random sample from the largest national online panel.

**Results:**

Compared with 2005 and 2010, the age at coital debut increased for both genders in 2021 (by a median of one year, to 18 years, and by a mean of half a year, to 17.5 years, in men and to 17.9 in women). In the 2005-2021 period, condom use increased by about 15% both at first intercourse (to 80%) and in consistent use (to 40% in women and 50% in men). When we controlled for basic socio-demographics, Cox and logistic regressions indicated that, for both genders, in 2005 and 2010 compared with 2021, the risks/odds were significantly higher for reporting an earlier sexual debut (adjusted hazard ratio 1.25-1.37), multiple sexual partners (adjusted odds ratio [AOR] 1.62-3.31), and concurrent relationships (AOR 3.36-4.64), while the odds were lower for condom use at first sexual intercourse (AOR 0.24-0.46) and consistently (AOR 0.51-0.64).

**Conclusion:**

Risky sexual behaviors decreased in the 2021 survey compared with the previous two waves, in both genders. Nonetheless, sexual risk-taking is still frequent among young Croatian adults. The introduction of sexuality education and other national-level public health interventions to reduce sexual risk-taking thus remains a public health imperative.

Sexual risk-taking refers to behaviors that can adversely affect reproductive, sexual, and psychological health, leading to sexually transmitted infections (STI), sexual victimization, unwanted pregnancies, and reduced well-being. Risky sexual behaviors typically include coital debut at an early age, unprotected sexual activities, having multiple sexual partners, and engaging in concurrent sexual relationships (1-3). Early sexual debut and having multiple partners are consistently associated with unprotected sex and an increased risk of acquiring an STI (4-7).

Globally, the highest age-specific prevalence of risky sexual behavior is found in adolescents and emerging adults, with condomless sexual intercourse ranking second among health-related mortality risks in young women and men (8). Transition to adulthood is a period of experimenting with various sexual practices and changing sexual partners (9). At the same time, young people often lack knowledge of sexual health, emotional and cognitive skills necessary for responsible decision-making, and communication competencies required for negotiating safe sex (9,10). The patterns of sexual risk-taking acquired at young age continue to affect sexual behaviors in later life, adding to the cumulative risk of STI (11).

Over the past 10-15 years, sexual activity and early sexual debut have somewhat decreased among young people internationally (12-19), albeit with significant socio-cultural variations. Nonetheless, the prevalence of STI, unplanned pregnancies, and abortions remained high or even increased among youth (5,10,12,13,17,20). In Europe, sexually active 15-24-year-olds bear the highest risk of *Chlamydia trachomatis* infection compared with all other age groups, with nearly half of new cases being observed in that group (21). Similarly, nearly 50% of all newly contracted common STI in the United States are found among adolescents and young adults (22).

In Croatia, findings from a sole national repeated cross-sectional study on sexual behavior among young people (conducted in 2005 and 2010) revealed substantial and stable levels of risky sexual behaviors (23-25). In particular, persistently low levels of consistent condom use (about 30%) were observed; one third of participants reported delayed condom application and one quarter reported concurrent sexual partners. At the same time, more than 80% of participants from both study waves believed that they were at low or no risk of contracting STI. More recently, poor knowledge of risks to sexual and reproductive health was reported among Croatian senior high-school students (26), as well as a high prevalence of human papillomavirus among young women (27). This hardly comes as a surprise due to the absence of school-based sexual education in the country – which remains a highly politicized issue – and the lack of organized national-level prevention efforts to reduce sexual risks (28-30).

Systematic monitoring of sexual behavior in Croatian youth is a public health imperative considering the levels of sexual risk-taking, the prevalence of common STI, and the lack of education in responsible sexual behavior. This article presents findings from the third wave of the national study on sexuality in young people in Croatia conducted in 2021. The study aimed to assess the current prevalence of risky sexual behaviors in emerging adults and their dynamics over the 2005-2021 period with the purpose of informing evidence-based national policy planning and public health interventions focused on young people’s sexual and reproductive health.

## Participants and methods

### Sampling procedure

In 2005 and 2010, face-to-face surveys on sexuality-related knowledge, attitudes, and behaviors were carried out on large-scale, national probability-based samples of young adults (24). The response rates were 79.5% in 2005 and 32.1% in 2010, and the non-response rates were 23.4% and 23.8%, respectively. The considerably lower response rate in 2010 corresponds to a long-term negative trend in survey participation (31,32). In 2021, the largest national commercial online panel was employed as the only feasible solution for data collection during the COVID-19 pandemic. Quota-based random sampling of the panel members was used with respect to region, age (18-25 cohort), and gender. After we applied *post-hoc* weighting for gender and age, the sample was broadly representative of the emerging adult population in Croatia. The response rate in the panel was 29%, with 84% of responders completing the survey. The socio-demographic structure of the three samples is presented in Table 1.

**Table 1:** Socio-demographic structure of the study samples by gender

	2005			2010			2021		
	women (n = 574)	men (n = 519)	total (n = 1093)	women (n = 495)	men (n = 510)	total (n = 1005)	women (n = 580)	men (n = 627)	total (n = 1207)
	n (%)	n (%)	N (%)	n (%)	n (%)	N (%)	n (%)	n (%)	N (%)
Father's education									
elementary school or less	80 (15.1)	51 (9.4)	131 (12.2)	45 (9.3)	41 (8.1)	86 (8.7)	78 (13.4)	45 (7.1)	122 (10.1)
high school	361 (68.1)	397 (73.1)	758 (70.6)	329 (67.7)	338 (66.7)	607 (67.2)	398 (68.6)	392 (62.5)	790 (65.4)
university degree	89 (16.8)	95 (17.5)	184 (17.2)	112 (23.0)	128 (25.2)	240 (24.2)	105 (18.0)	191 (30.4)	295 (24.5)
Mother's education									
elementary school or less	85 (15.9)	105 (19.0)	190 (17.5)	62 (12.5)	59 (11.6)	121 (12.1)	68 (11.8)	44 (7.1)	112 (9.3)
high school	348 (65.2)	333 (60.2)	681 (62.7)	334 (67.5)	348 (68.4)	682 (67.9)	388 (66.9)	386 (61.5)	774 (64.1)
university degree	101 (18.9)	115 (20.8)	216 (19.8)	99 (20.0)	102 (20.0)	201 (20.0)	123 (21.3)	183 (29.3)	321 (26.6)
Family socioeconomic status									
lower than average	49 (9.1)	42 (7.6)	91 (8.4)	16 (3.2)	21 (4.1)	37 (3.7)	75 (12.8)	58 (9.4)	133 (11.0)
about average	382 (71.3)	391 (71.1)	773 (71.2)	367 (74.1)	365 (71.6)	732 (72.8)	385 (66.3)	385 (61.4)	770 (63.8)
higher than average	105 (19.6)	117 (21.3)	222 (20.4)	112 (22.6)	124 (24.3)	236 (23.5)	121 (20.9)	183 (29.2)	304 (25.2)
Attendance of religious services									
never or non-religious	130 (24.2)	174 (31.6)	304 (28.0)	147 (29.8)	189 (37.1)	336 (33.5)	110 (19.0)	159 (25.4)	270 (22.4)
up to several times a year	182 (33.9)	211 (38.4)	393 (36.2)	185 (37.4)	191 (37.5)	376 (37.5)	306 (52.6)	321 (51.1)	626 (51.8)
once a month	115 (21.4)	75 (13.6)	190 (17.5)	87 (17.6)	66 (12.9)	153 (15.2)	59 (10.2)	56 (8.9)	115 (9.5)
once a week or more	110 (20.5)	90 (16.4)	200 (18.3)	75 (15.2)	64 (12.5)	139 (13.8)	105 (18.2)	91 (14.6)	197 (16.3)
Settlement of longest residence by size									
≤10 000 inhabitants	259 (48.9)	305 (55.2)	564 (52.1)	249 (50.4)	256 (50.5)	505 (50.4)	334 (57.5)	291 (46.4)	625 (51.8)
>10 000 inhabitants	315 (51.1)	214 (44.8)	529 (47.9)	246 (49.6)	254 (49.5)	500 (49.6)	246 (42.5)	336 (53.6)	582 (48.2)

### Participants

In 2005, the sample included 1092 participants in the 18-24 age-range (*M*_age_ = 21.0, 49.3% female). In 2010 and 2021, the age ranged from 18-25 years. In 2010, the sample included 1005 participants (*M*_age_ = 21.5, 49.3% female) and in 2021 – 1210 (*M*_age_ = 21.7, 48.0% female). The one-year difference in age ranges did not compromise between-study comparisons. All but one out of the 1764 sexually active participants from the 2010 and 2021 samples reported having had the first sexual intercourse under the age of 25. Furthermore, 25-year-olds in 2010 and 2021 did not differ from 24-year-olds in the reported lifetime number of sexual partners (*χ^2^_2010_* = 8.96, *P* = 0.11; *χ^2^_2021_* = 6.94, *P* = 0.23), the number of partners in the past year (*χ^2^_2010_* = 7.17, *P* = 0.10; *χ^2^_2021_* = 0.98, *P* = 0.81), condom use at first intercourse (*χ^2^_2010_* = 0.89, *P* = 0.35; *χ^2^_2021_* = 0.04, *P* = 0.84), and consistent condom use in the past year (*χ^2^_2010_* = 5.01, *P* = 0.08; *χ^2^_2021_* = 1.04, *P* = 0.59). Three participants from the 2021 sample who provided impossible values (eg, being 55 at coital debut) were excluded from the final sample (N_2021_ = 1207).

### Data collection

In 2005 and 2010, participants were interviewed in their homes. In 2021, computer-assisted web-interviewing was used. Members of the commercial online panel were selected randomly. Data were collected from mid-November 2021 to early January 2022. The study was approved by the Ethics Review Board of the Faculty of Humanities and Social Sciences, University of Zagreb. All participants gave informed consent verbally or electronically. The 2010 and 2021 surveys additionally included a biological component (not reported here).

### Questionnaire

An originally developed questionnaire on knowledge, attitudes, beliefs, and practices was used. The first part (administered by face-to-face interviewing in 2005 and 2010) asked about socio-demographic characteristics, HIV/AIDS knowledge, attitudes toward gendered sexual roles, beliefs about condom use, and self-esteem. The rest was self-administered and focused on sexual behaviors. The questionnaires consisted of 150-190 items and took 25-30 minutes to complete. The original questionnaire was piloted among high-school and university students. The instruments used for comparisons were identical in all study waves.

### Measures

Age, parental education, family socio-economic status, the type of the longest place of residence, and the frequency of attending religious services were assessed as socio-demographic characteristics (Table 1). Risky sexual behaviors were assessed with the following one-item indicators: age at sexual debut (defined as first coital intercourse), protection/contraception use at first sexual intercourse, condom use consistency (past 12 months), the number of sexual partners (“individuals you had vaginal intercourse with”) over a lifetime and during the last 12 months, and ever having concurrent sexual relationships. An additional socio-sexual characteristic measured was the gender of sexual partners (“persons with whom you had oral, anal, or vaginal sex”).

### Statistical analysis

Survival analysis was carried out to compare coital debut among study waves. The probability of (not) having coitus before a certain age was estimated by Kaplan-Meier curves. The significance of the between-study difference in sexual initiation was assessed with a log-rank test. Cox regression was used to assess changes in sexual initiation when controlling for basic socio-demographic characteristics. To assess change over time in other core indicators of sexual risk-taking (the number of sexual partners in the past 12 months, condom use at first intercourse, consistent condom use, and concurrent sexual relationships), multivariate binary logistic regression analysis was employed with socio-demographics controlled for. All statistical analyses were performed with SPSS Statistics, version 25 (IBM Corp., Armonk, NY, USA). The probability value <0.05 was set as a threshold for statistical significance.

## Results

### Sexual experiences and behaviors over the 2005-2021 period

About 84% female and 88% male participants from the 2005 and 2010 samples experienced sexual intercourse (Table 2). In 2021, the proportion decreased by almost 10% in women and by 20% in men. This change was reflected in the age at sexual debut. Compared with earlier study waves, in 2021 the average age at sexual debut increased by about half a year, to 17.5 years (standard deviation [SD] 2.3) in men (95% confidence interval [CI] 17.3-17.7) and to 17.9 (SD 1.7) in women (95% CI 17.7-18.1). Similarly, the median age at sexual debut increased from 17 years (interquartile range [IQR] 16-18) for both genders in 2005 and 2010 to 18 years in 2021 (IQR 17-19 for women and 16-19 for men).

**Table 2:** Sexual behaviors, experiences, and patterns of condom use by study year and gender*

	Women			Men		
	2005 (n = 574)	2010 (n = 495)	2021 (n = 580)	2005 (n = 519)	2010 (n = 510)	2021 (n = 627)
	% (95% CI^†^)	% (95% CI)	% (95% CI)	% (95% CI)	% (95% CI)	% (95% CI)
Experience of sexual intercourse	82.7 (79.5-85.9)	84.4. (81.2-87.6)	76.3 (72.8-79.8)	87.7 (85.00-90.4)	87.9 (85.1-90.7)	69.4 (65.8-73.00)
Age of coital debut (years)						
≤15	7.5 (5.0-10.0)	10.7 (7.7-13.7)	5.2 (3.1-7.3)	14.6 (11.4-17.8)	20.2 (16.5-24.0)	12.4 (9.3-15.5)
16	20.0 (16.2-23.8)	17.5 (13.8-21.2)	16.5 (13.0-20.0)	20.9 (13.2-24.6)	22.9 (19.0-26.8)	14.3 (11.0-17.6)
17	27.2 (23.0-31.4)	26.5 (22.2-30.8)	22.5 (18.6-26.4)	26.7 (22.7-30.8)	22.7 (18.8-26.6)	22.9 (19.0-26.8)
18	15.4 (12.0-18.8)	23.8 (19.7-27.9)	24.0 (20.0-28.0)	24.3 (20.4-28.2)	19.5 (15.8-23.2)	24.8 (20.8-28.8)
≥19	30.0 (25.7-34.4)	21.4 (17.4-25.4)	31.7 (27.4-36.0)	13.5 (10.4-16.6)	14.7 (11.4-18.0)	25.6 (21.5-29.7)
Number of sexual partners (lifetime)						
1	35.5 (30.9-40.1)	29.5 (24.1-32.9)	38.0 (33.5-42.5)	14.6 (11.2-18.0)	14.6 (11.2-18.0)	33.0 (25.7-34.3)
2-3	25.8 (22.2-29.4)	29.3 (25.3-33.3)	28.0 (24.4.-31.7)	32.6 (28.6-36.6)	22.9 (19.3-26.6)	33.3 (29.6-36.7)
4-5	17.9 (14.2-21.6)	18.7 (14.9-22.5)	13.3 (10.1-16.5)	21.2 (17.3-25.1)	21.0 (17.1-25.0)	17.9 (14.3-21.5)
6-9	8.9 (6.2-11.7)	8.1 (5.5-10.8)	8.8 (6.2-11.4)	15.0 (11.6-18.4)	19.5 (15.7-23.3)	9.2 (6.5-11.9)
≥10	4.5 (2.2-6.5)	6.9 (4.4-9.4)	4.9 (2.9-6.9)	16.5 (13.0-20.0)	22.0 (18.0-26.0)	9.6 (6.8-12.4)
Number of sexual partners (past year)						
0	9.1 (6.4-11.8)	4.2 (2.3-6-1)	7.0 (4.6-9.4)	9.3 (6.6-12.0)	5.3 (3.2-7.4)	9.9 (7.1-12.7)
1	68.8 (64.4-73.2)	72.9 (68.6-77.2)	79.1 (75.3-82.9)	48.1 (43.5-52.7)	49.1 (44.4-53.8)	70.1 (65.8-74.4)
2-3	19.3 (15.5-23.1)	19.1 (15.3-22.9)	12.4 (9.9-15.5)	30.9 (26.7-35.1)	28.6 (24.3-32.9)	15.9 (12.5-19.3)
≥4	2.9 (1.5-4.5)	3.9 (2.0-5.8)	1.5 (0.4-2.6)	11.7 (8.8-14.6)	17.0 (13.5-20.6)	4.0 (2.2-5.8)
Concurrent sexual relationship (ever)	16.5 (13.0-20.0)	17.2 (13.6-20.8)	4.8 (2.8-6.8)	31.1 (26.9-35.3)	29.0 (24.8-33.2)	11.1 (8.2-14.1)
Exclusively opposite-sex sexual partners	92.5 (90.0-95.0)	93.2 (90.8-95.6)	95.5 (93.6-97.4)	93.2 (90.9-95.5)	95.0 (93.0-97.0)	94.0 (91-8-96.2)
Condom use at first sexual intercourse	57.7 (53.1-62.3)	71.4 (67.1-75.7)	83.5 (80.0-87.0)	62.8 (58.9-67.1)	65.8 (61.4-70.2)	81.1 (77.4-84.8)
Consistent condom use (past year; only those who had sex during the past year)	(n = 314) 25.9 (21.1-30.8)	(n = 319) 29.8 (24.8-34.8)	(n = 317) 39.7 (34.3-45.1)	(n = 310) 34.0 (28.7-39.3)	(n = 322) 32.0 (26.9-37.1)	(n = 334) 47.4 (42.1-52.8)

*Only participants who reported sexual intercourse were included.

†CI – confidence interval.

**Figure 1:**
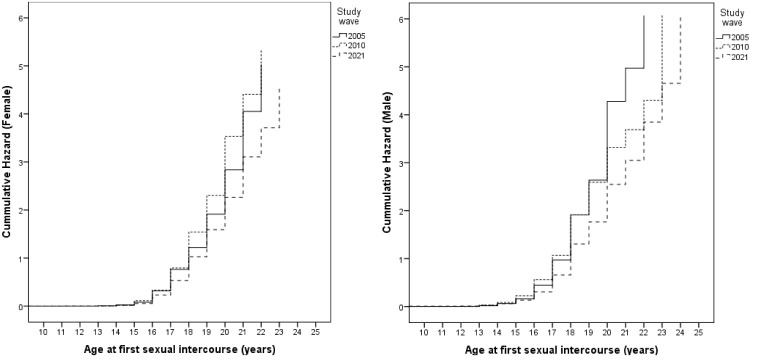
Hazard function of sexual initiation in survival analysis indicates an increase in age at sexual debut in both genders in the 2005-2021 period (only participants who reported sexual intercourse were included).

Kaplan-Meier’s estimation of the hazard function of sexual initiation also indicated an increase in age at sexual debut (Figure 1). Log-rank testing confirmed that the change was significant for both women (M_(2)_ = 41.68, *P* < 0.001) and men (M_(2)_ = 34.94, *P* < 0.001).

The lifetime number of sexual partners was three or fewer for most young women across the observed period (Table 2), with a median of two in 2005 and 2021 and three in 2010 (IQR 1-4 in all cases). The change in the number of sexual partners was more pronounced in young men. While considerable proportions of male participants in the 2005 and 2010 samples reported more than five (around 30%) or even more than 10 (16.5%-22%) lifetime sexual partners, the majority of men in 2021 (around 63%) reported up to three partners. The proportion of those who had a single lifetime partner more than doubled, from about 15% in 2005 and 2010 to 33% in 2021. Accordingly, the median number of men’s sexual partners decreased from four (IQR 2-7) in 2005 and five (IQR 3-8) in 2010 to three (IQR 1-5) in 2021. As for the past year, the majority of female participants (69%-79%) reported a single partner. Among men, one sexual partner was reported by the majority of participants in all study waves as well, with this proportion increasing from about 50% in 2005 and 2010 to 70% in 2021.

In all study waves, roughly twice as many men as women reported ever having concurrent sexual partnerships (Table 2). However, the proportion of participants reporting the experience decreased by a factor of three in both women (from about 17% in 2005 and 2010 to 5% in 2021) and men (from about 30% in 2005 and 2010 to 11% in 2021).

In 2021, condom use at first intercourse was reported by over 80% of female and male participants (Table 2), an increase from the previous wave of about 12% in female and 15% in male participants. Consistent condom use over the past 12 months among the sexually active participants increased by 10%-15% in 2021 compared with previous waves. Even with this increase, less than 50% of male and 40% of female participants reported consistent use of condoms in 2021.

### Change in sexual risk-taking in the 2005-2021 period

Next, we assessed temporal changes in the select five indicators of sexual risk-taking (age at coital debut, the number of sexual partners in the past year, condom use at first vaginal intercourse, consistent condom use over the past year, and concurrent sexual relationships) while accounting for basic socio-demographic characteristics. Cox regression indicated that the risk of reporting earlier sexual debut was significantly higher in men in 2005 and 2010 compared with 2021 (by 1.25-1.37 times, respectively), and in women by 1.27 times in 2010 compared with 2021 (Table 3).

**Table 3:** Cox regression with age at first sexual intercourse as outcome, study wave as a predictor, and basic socio-demographic characteristics as controls, by gender*^†^

	Women	Men
	AHR (95% CI)	AHR (95% CI)
Study wave		
2005	1.12 (0.98-1.28)	1.25 (1.09-1.44)^‡^
2010	1.27 (1.11-1.46)^‡^	1.37 (1.19-1.57)§
2021 (referent)	1	1
Age	0.88 (0.85-0.90)^§^	0.89 (0.87-0.92)^§^
Father’s education		
elementary school	0.88 (0.70-1.11)	1.12 (0.87-1.45)
high school	0.90 (0.77-1.04)	1.10 (0.95-1.27)
university (referent)	1	1
Mother’s education		
elementary school	1.01 (0.81-1.25)	1.16 (0.92-1.46)
high school	1.00 (0.86-1.16)	1.05 (0.91-1.22)
university (referent)	1	1
Family socioeconomic status	1.02 (0.93-1.12)	0.99 (0.90-1.09)
Attendance of religious services	0.91 (0.88-0.94)^§^	0.96 (0.93-0.99)^||^
Settlement of longest residence by size		
≤10 000 inhabitants	1.00 (0.90-1.12)	1.12 (0.99-1.26)
>10 000 inhabitants (referent)	1	1

* Abbreviations: AHR – adjusted hazard ratio; CI – confidence interval.

†Only participants who reported sexual intercourse were included.

‡*P* < 0.01.

§*P* < 0.001.

||*P* < 0.05.

Similarly, the odds of reporting any of the four remaining core indicators of sexual risk-taking - when we controlled for socio-demographic characteristics - were lower in 2021 compared with previous surveys, regardless of gender (Table 4). Female and male participants in 2005 and 2010 were 1.62-3.31 times more likely to report multiple sexual partners in the past year and 3.36-4.64 times more likely to report ever having concurrent sexual partners than in 2021. Compared with 2021, in 2005 and 2010 both genders were less likely to report condom use at first sexual intercourse (by 54%-76%) and consistently over the past 12 months (by 36%-49%).

**Table 4:** Binary logistic regressions with sexual partners in the past year, condom use at first sexual intercourse, consistent condom use in the past year, and concurrent sexual relationships as outcomes, study wave as predictor, and basic socio-demographics as controls, by gender*^†^

	Sexual partners (past year) (0 = 0 and 1; 1 = 2 or more)	Condom use at first sexual intercourse (0 = no; 1 = yes)	Consistent condom use (past year) (0 = no; 1 = yes)	Concurrent sexual relationships (ever) (0 = no; 1 = yes)

women	men	women	men	women	men	women	men
AOR (95% CI)^†^	AOR (95% CI)	AOR (95% CI)	AOR (95% CI)	AOR (95% CI)	AOR (95% CI)	AOR (95% CI)	AOR (95% CI)
Study wave								
2005	1.62 (1.11-2.34)^||^	2.82 (2.07-3.85)^§^	0.24 (0.18-0.34^§^	0.36 (0.26-0.49)^§^	0.51 (0.36-0.72)^§^	0.55 (0.40-0.77)^‡^	4.64 (2.74-7.85)^§^	3.92 (2.71-5.67)^§^
2010	1.70 (1.18-2.45)^§^	3.31 (2.43-4.51)^§^	0.46 (0.33-0.65)^§^	0.44 (0.32-0.60)^§^	0.64 (0.46-.0.90)^||^	0.54 (0.39-0.75)^§^	4.54 (2.69-7.65)^§^	3.36 (2.32-4.85)^§^
2021 (referent)	1	1	1	1	1	1	1	1
Age	0.87 (0.81-.94)^§^	0.89 (0.84-0.94)^§^	0.91 (0.88-0.97)^‡^	0.87 (0.82-0.93)^§^	0.90 (0.84-0.96)^‡^	0.87 (0.81-0.93)^§^	0.97 (0.89-1.06)	1.04 (0.97-1.10)
Father’s education								
elementary school	1.00 (0.54-1.84)	0.98 (0.56-1.70)	0.55 (0.33-0.93)^||^	0.75 (0.43-1.30)	0.60 (0.33-1.08)	0.75 (0..37-1.50)	1.79 (0.89-3.61)	0.68 (0.36-1.30)
high school	1.06 (0.72-1.56)	0.77 (0.56-1.04)	0.93 (0.64-1.34)	1.01 (0.73-1.39)	0.82 (0.56-1.20)	1.07 (0.76-1.51)	0.82 (0.51-1.30)	1.02 (0.73-1.44)
university (referent)	1	1	1	1	1	1	1	1
Mother’s education								
elementary school	0.93 (0.52-1.68)	1.27 (0.79-2.05)	0.70 (0.42-1.16)	0.87 (0.55-1.43)	0.97 (0.55-1.71)	0.71 (0.40-1.26)	0.43 (0.19-0.98)^||^	1.55 (0.92-2.62)
high school	0.96 (0.65-1.40)	1.17 (0.85-1.61)	0.88 (0.62-1.26)	1.01 (0.73-1.39)	1.00 (0.68-1.48)	0.96 (0.67-1.36)	1.24 (0.78-1.98)	1.06 (0.75-1.51)
university (referent)	1	1	1	1	1	1	1	1
Family socioeconomic status	1.54 (1.21-1.97)^‡^	1.01 (0.82-1.24)	1.11 (0.90-1.37)	1.33 (1.08-1.65)^‡^	1.10 (0.87-1.39)	0.83 (0.66-1.05)	1.20 (0.89-1.60)	1.15 (0.91-1.45)
Attendance of religious services	0.84 (0.76-0.93)^‡^	0.93 (0.86-1.01)	0.99 (0.91-1.08)	1.02 (0.94-1.11)	0.96 (0.87-1.06)	1.02 (0.93-1.12)	0.78 (0.70-0.88)^§^	0.88 (0.80-0.96)^‡^
Settlement of longest residence by size								
≤10 000 inhabitants	1.41 (1.05-1.90)^||^	0.97 (0.76-1.24)	1.03 (0.79-1.34)	1.10 (0.86-1.41)	0.93 (0.70-1.25)	0.87 (0.66-1.15)	0.97 (0.68-1.38)	1.06 (0.81-1.39)
>10 000 inhabitants (referent)	1	1	1	1	1	1	1	1

*Abbreviations: AOR – adjusted odds ratio; CI – confidence interval.

†Only participants who reported sexual intercourse were included.

‡*P* < 0.01.

§*P* < 0.001.

||*P* < 0.05.

Overall, the observed associations between study wave and sexual risks were moderate in size in the case of concurrent sexual relationships, condom use at first intercourse, and the number of sexual partners in the past 12 months. In other instances, the effect was small (33).

## Discussion

A previous comparison of the 2005 and 2010 surveys pointed to stable and substantial levels of sexual risk-taking (24). In the 2021 survey, nearly all core sexual risk indicators significantly decreased compared with the 2005-2010 period. However, the prevalence of sexual risk-taking among young people in Croatia remains substantial, particularly the prevalence of inconsistent condom use.

The observed change can be attributed to at least two sets of factors. The first one is related to the COVID-19 pandemic considering that the 2021 wave was conducted 20-22 months after its onset. The imposed restrictions – as well as a spillover effect of sensitization to health-related risks from the COVID-19 infection (34,35) – may have affected sexual behaviors measured in the retrospect of 12 months (fewer sexual partners, more consistent condom use) (34,36-38). A delayed coital debut and a decreased number of sexual partners in younger participants are also possible. Nevertheless, it is unlikely that the change observed in this study is predominantly associated with the pandemic. Most young people did not stop engaging in sexual risk-taking during the pandemic (39-41), some engaging even more intensely so as to counter stress and loneliness (42). Additionally, except in the initial two months of the pandemic, the Croatian government imposed a soft lockdown (43), not limiting within-country movement and social contact in small groups. In a national online survey of emerging Croatian adults’ intimacy and sexuality during the COVID-19 pandemic, conducted 10 months following its outbreak (44), only 3% of participants reported pandemic-related separation from their partners. Additionally, an equal proportion of sexually active participants (12%) reported having no sexual partners or having multiple sexual partners during the pandemic. A lower frequency of condom use during the pandemic was reported by 16% of participants, while only 8% reported an increased frequency of condom use. To summarize, it appears that the pandemic affected the sex lives of a minority of emerging Croatian adults and did so inconsistently, contributing both to less and more sexual risk-taking.

The observed change in sexual risk-taking seems likelier to have been influenced by a second, socio-cultural, set of factors. International studies carried out before the pandemic pointed to a declining trend in sexual activity among younger cohorts in industrialized countries (12-19). Decreased sexual activity, delayed coital debut, and a lower number of sexual partners are attributed to several recent developments. Ubiquitous social media use among young people (45,46) could be a partial replacement or even a barrier for real-life sexual interactions given that online social networking provides new forms of sexual expression (47), increases insecurity regarding physical appearance and sexual performance (48,49), supplies entertainment that may compete with sexual activity (15) (such as video streaming services and online sexual contents) (50), and impedes the development of face-to-face communication skills (51,52). Another possible explanation is delayed transition to adulthood (50,53,54), which involves postponed partnered cohabitation and prolonged co-residence with parents. This entails reduced opportunities for sexual activity and persistent parental control over behaviors that often contribute to sexual risk-taking, such as substance abuse (52). There is also increasing evidence about the effectiveness of comprehensive school-based sex education in promoting condom use, which reduces the risk of acquiring STI, and helps managing potentially harmful effects of sexualized media (13,55-57). Finally, religiosity has been on the rise among young people in the West over the past 15-20 years, a process partially influenced by neo-conservative social and political movements (58). Although religiosity provides limited protection against sexual risk-taking among young people (59,60), intrinsic religiosity is increasing in Croatia (61), with the Catholic church and newly established faith-based civic associations actively encouraging young people to practice and advocate religious values (62,63).

The present study has several limitations. First, the validity of the findings reported across the three surveys is limited by self-reporting and some indicators are additionally affected by recall bias. However, such bias is likely limited, because our participants were in early stages of their sexual lives, and the retrospective measures of sexual behavior were limited to the past 12 months. Additionally, there is no alternative to self-reporting when exploring sexual behaviors such as lifetime number of sexual partners or consistency in condom use. We attempted to minimize social desirability in participants’ responses to sensitive questions in the 2005 and 2010 face-to-face surveys by employing experienced interviewers who received an additional six-hour training focused on collecting information on sexuality-related topics. Privacy was secured by measuring sexual behaviors and experiences with a self-administered questionnaire.

Second, health-related and social context surrounding the COVID-19 pandemic may have affected the sexual behavior of young Croatian adults reported in the 2021 survey and reduced comparability with previous surveys. As previously discussed, if present, this effect is unlikely to have substantially affected the findings considering that the lockdown in Croatia was mild, and emerging adults reported either no effect of the pandemic on their sex lives or reported mixed outcomes (44).

Third, another potential obstacle to between-wave comparisons was the diverging sampling and data collection approach between the 2021 and the 2005 and 2010 studies. The national online panel was used to facilitate data collection during the pandemic, but also to tackle the substantial and continued decrease in response rates observed in conventional field surveys (64), particularly among young people (65). Large self-selection bias calls into question the probabilistic nature of conventional (probability-based) samples, but also data comparison between studies conducted with matching methodologies (66). Additionally, certain drawbacks raised in the context of commercial online samples, such as that participants are “professionalized,” were shown not to substantially affect data quality (67). It also needs to be reiterated that in 2005 and 2010 sexual behaviors and experiences - including the core indicators of sexual risk-taking - were measured with a self-administered (paper-and-pencil) questionnaire, a method comparable to self-administered online surveying employed in 2021. Finally, a data harmonization procedure (68) was performed using the 2010 and 2021 surveys to empirically assess the comparability of the data obtained by different sampling and gathering strategies (not reported here). The correlation of correlations test involving single-item measures of sexual behavior, including the core five indicators of sexual risk-taking, suggested adequate within- and between-study construct validity of the measures, justifying the direct data comparisons in the current study (69).

In spite of these shortcomings, the three surveys remain the sole national-level research project in Croatia aimed at monitoring and analyzing sexual behaviors, attitudes, and beliefs among emerging adults.

In conclusion, sexual risk-taking is still relatively frequent among young people in Croatia, but has substantially declined in the past decade. This positive change does not appear to be driven by any systematic efforts to reduce sexual and reproductive health risks. At the national level, public health efforts to improve sexual and reproductive health in young people remain sporadic and lacking in evidence about their efficacy. Due to political controversies, comprehensive sexuality education is not included in the national educational curriculum despite the fact that the majority of young people (70) and their parents (71) are in favor of such an addition. Continuous monitoring of sexual behaviors in young people therefore remains a public health imperative, as do continued efforts in providing evidence-based prevention, intervention, and systematic education aimed at improving young people’s sexual and reproductive health. Findings from this study should be considered when developing sexual health counseling programs delivered by school medicine specialists, who provide preventive health services to school-aged children and university students. Additionally, digital media interventions should be designed to promote sexual health, as such programs reach large audiences at low cost and provide anonymity and privacy for the users (72).
